# Dynamics of microbial contamination and hygiene risk points in the production of ready-to-eat Yao meat

**DOI:** 10.3389/fmicb.2026.1829503

**Published:** 2026-04-22

**Authors:** Minjun Liu, Yaowen Zhang, Xia Chen, Fangzhe Ren, Mingshu Xu, Honggang Lai

**Affiliations:** 1 School of Tourism and Cuisine, Yangzhou University, Yangzhou, China; 2 Key Laboratory of Chinese Cuisine Intangible Cultural Heritage Technology Inheritance, Ministry of Culture and Tourism, Yangzhou, China; 3 Jiangsu Key Lab of Zoonosis, Yangzhou University, Yangzhou, China; 4 Key Laboratory of Prevention and Control of Biological Hazard Factors (Animal Origin) for Agri-food Safety and Quality, Ministry of Agriculture of China, Yangzhou, China; 5 Zhejiang Meat Processing and Quality Control Engineering Technology Research Center, Zhejiang, China

**Keywords:** 16S rRNA sequencing, hygiene risk points, microbial contamination dynamics, ready-to-eat meat, source tracking, Yao meat

## Abstract

Ready-to-eat (RTE) meat products processed under mild or low-temperature conditions are particularly susceptible to microbial contamination. In this study, culture-based methods combined with 16S rRNA gene sequencing were applied to investigate microbial contamination dynamics and identify hygiene risk points throughout the production of Yao meat in a central kitchen in eastern China. The highest microbial loads were observed during the thawing stage, with total viable counts reaching 6.08 ± 0.30 log_10_ CFU/cm^2^ and coliform counts of 3.35 ± 0.57 log_10_ CFU/cm^2^. Although cooking significantly reduced microbial levels, a marked increase during the packaging stage indicated post-processing contamination associated with inadequate hygiene control. High relative abundances of potentially spoilage indicator taxa, including *Chryseobacterium*, *Campylobacter*, and *Pseudomonas*, were detected during the kneading stage, suggesting their contribution to downstream contamination. Source tracking analysis revealed that approximately 78.32% of the microbial community in the final product originated from the thawing and kneading stages. In contrast, *Bacillus* spp. dominated the end product without a clearly identifiable source, indicating the presence of uncharacterized contamination routes. Overall, these results identify critical hygiene risk points during Yao meat production and provide insights into microbial contamination patterns in traditional RTE meat products.

## 1 Introduction

Foodborne diseases remain a major global health threat, causing an estimated 600 million illnesses and 420,000 deaths annually (World Health Organization [WHO], 2020). Ready-to-eat (RTE) meat products are widely consumed for their convenience but are highly susceptible to microbial contamination during production and distribution (Bodie et al., 2023). Although RTE meat is sterilized by heating, the product may be contaminated again in the subsequent cutting, packaging, transportation and other links due to incomplete disinfection of equipment, operators’ hands or tools carrying pathogenic bacteria (Yang et al., 2016). RTE meats are recognized as high-risk carriers of pathogens such as Listeria monocytogenes (Zambon et al., 2022; Zhang et al., 2021). Since RTE food can be eaten without further processing, they have been shown to be more susceptible to microbial contamination compared with other types of meats products (Baloch et al., 2017), leading to foodborne illness outbreaks (Ji et al., 2015; Wang et al., 2020). Therefore, rigorous microbial control measures are essential across the production chain to ensure consumer safety.

Yao meat, a traditional salted and stewed RTE meat product, is valued for its unique flavor (Yao et al., 2018). It is made by pickling pork in spices, simmering it gently, then refrigerating and pressing the product to promote broth gelation and shape-setting, thus yielding a unique jelly texture and rich flavor. However, the product needs to be molded by low-temperature pressing after cooking, which cannot further reduce the microbial load through secondary high-temperature sterilization. At present, research on microbial contamination of RTE meat products at home and abroad mainly focuses on Western style ham, sausages and other products, and there is still a lack of specialized prevention and control technologies for the industrial production of traditional Chinese meat products. The current research on Yao meat is limited to describing the microbial community structure after packaging and results have shown that *Bacillus* dominates the microbial composition of the final product, lacking a systematic analysis of the dynamics throughout the entire production chain (Zhang et al., 2018).

Compared with traditional techniques, 16S rRNA gene sequencing provides a more accurate and efficient method for microbial identification. This method has been successfully applied to characterize microbial dynamics in preserved meats such as bacon (Li et al., 2021) and during the storage of various meat dishes (Xiao et al., 2013). However, for RTE meat, most studies to date have focused on the preservation of products, leaving a gap in understanding microbial community structures across the entire RTE meat production process.

This study used high-throughput 16S rRNA gene sequencing to investigate the microbial community composition during the production process of Yao meat from raw material processing to final packaging. The findings identified the potential contamination points for microbial contamination, elucidated the dominant microbial taxa at each processing stage, and provided targeted insights for Yao meat production, but also for those in the broader field of RTE meat production.

## 2 Materials and methods

### 2.1 Sample collection

**FIGURE 1:**
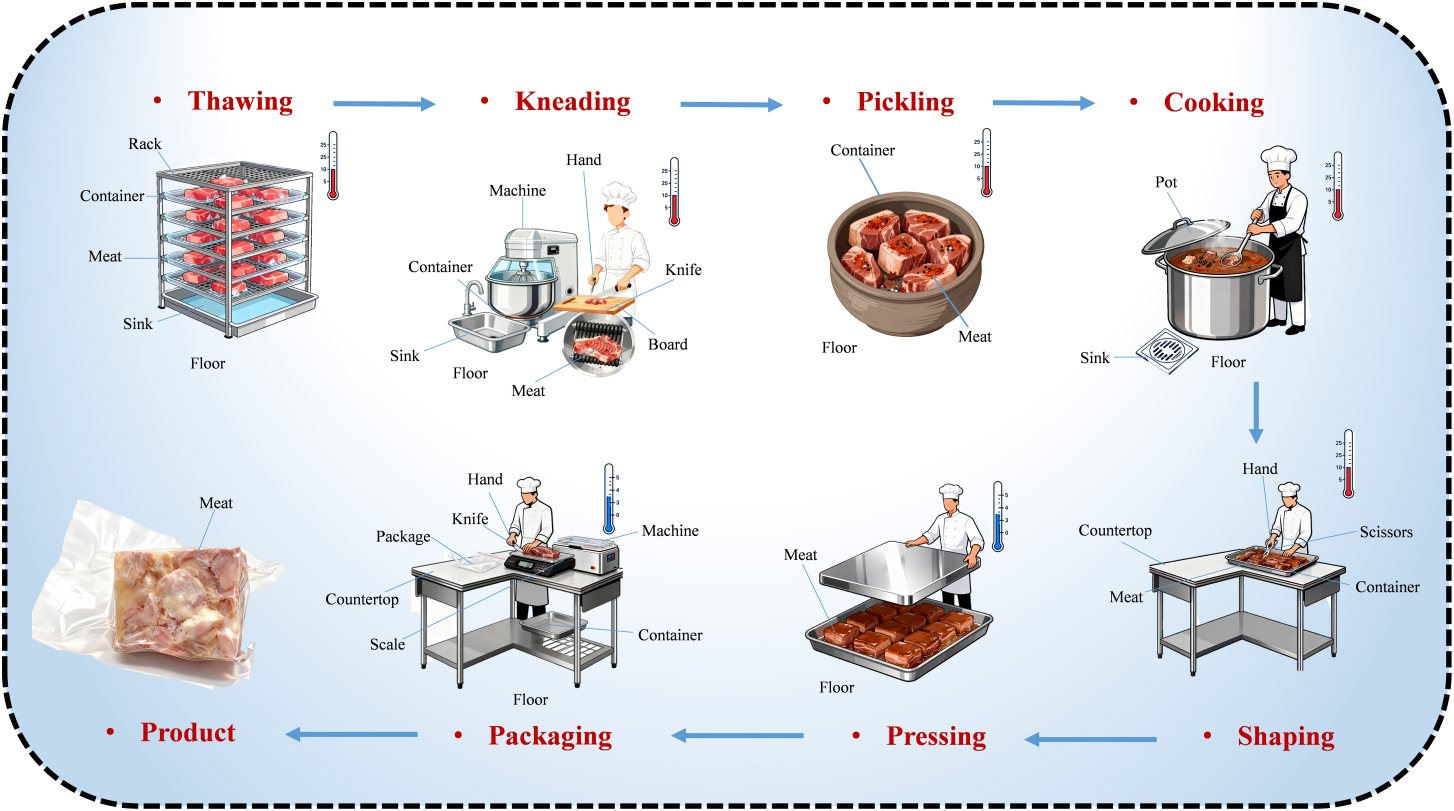
Illustration of the sampling sites and products in Yao meat line.

All samples were collected in December 2024 from a central kitchen in eastern China that specializes in the production of Yao meat (Figure 1). A total of 130 samples were obtained, including 95 environmental samples collected across seven production stages and 35 samples of the meat samples. Uneven sample sizes across stages and matrices reflected production rhythm, stage scale, and sample availability during continuous industrial processing. For each sampling surface, two sterile swabs prewetted with phosphate buffered saline (PBS) were used to wipe an area of 100 cm^2^ and placed into a sterile sampling bag. Sampling sites included various contact surfaces, such as racks, sinks, floors, containers, machines, pots, scissors, countertops, hands, scales, and knives. For meat samples, 250 g of each specimen was collected and placed into a sterile sampling bag. All samples were transported to the laboratory under aerobic condition at 4°C and analyzed within 12 h of collection. Refer Table 1 for specific sample size of each sampling site.

**TABLE 1:** Sanitary conditions of samples in each technological process of Yao meat production.

Category	Processing	Sampling location or sample	Microbial count (log_10_ CFU/cm^2^)/(log_10_ CFU/g)		
			Total viable counts	Coliform	*E. coli*
Production facilities and environment	Thawing	Rack (*n* = 3)	5.71 ± 0.57^a^	2.45 ± 0.60^ab^	ND
		Sink (*n* = 3)	6.08 ± 0.30^a^	3.35 ± 0.57^a^	ND
		Floor (*n* = 4)	4.90 ± 1.33^ab^	2.92 ± 0.78^ab^	1.43 ± 0.00
		Container (*n* = 3)	3.08 ± 0.26^b^	1.74 ± 0.18^b^	ND
	Kneading	Machine (*n* = 6)	4.90 ± 1.11^a^	1.49 ± 0.94^ab^	1.00 ± 0.00
		Board (*n* = 3)	3.45 ± 0.10^b^	2.02 ± 0.02^a^	ND
		Knife (*n* = 3)	ND	1.00 ± 0.12^ab^	ND
		Hand (*n* = 3)	2.88 ± 0.10^b^	0.48 ± 0.02^b^	ND
		Floor (*n* = 5)	3.82 ± 0.62^ab^	2.33 ± 0.66^a^	0.60 ± 0.00
		Sink (*n* = 3)	3.91 ± 0.54^ab^	2.20 ± 0.65^a^	ND
		Container (*n* = 3)	4.93 ± 0.49^a^	1.36 ± 0.00^ab^	ND
	Pickling	Container (*n* = 5)	3.64 ± 1.05	3.15 ± 0.90	1.53 ± 0.27
		Floor (*n* = 3)	4.51 ± 1.98	1.50 ± 1.13	ND
	Cooking Shaping	Sink (*n* = 3)	2.91 ± 0.29^a^	1.33 ± 0.25^a^	ND
		Floor (*n* = 3)	3.49 ± 0.91^a^	1.33 ± 0.18^a^	ND
		Pot (*n* = 3)	ND	0.78 ± 0.13^a^	ND
		Scissors (*n* = 3)	3.61 ± 0.03^a^	0.48 ± 0.07^c^	ND
		Countertop (*n* = 3)	3.72 ± 0.02^a^	1.79 ± 0.11^b^	ND
		Container (*n* = 3)	4.21 ± 0.43^a^	3.12 ± 0.34^a^	1.20 ± 0.00
		Hand (*n* = 3)	ND	ND	ND
	Pressing	Floor (*n* = 3)	3.23 ± 0.04	2.58 ± 0.05	ND
	Packaging	Floor (*n* = 3)	3.98 ± 0.06^a^	3.37 ± 0.78^a^	ND
		Hand (*n* = 3)	4.10 ± 0.56^a^	3.14 ± 0.55^a^	ND
		Machine (*n* = 3)	4.09 ± 0.05^a^	3.23 ± 0.78^a^	ND
		Scale (*n* = 3)	3.97 ± 0.12^a^	2.74 ± 0.09^a^	ND
		Container (*n* = 3)	4.09 ± 0.67	3.32 ± 0.24^a^	ND
		Knife (*n* = 3)	4.38 ± 0.34^a^	3.51 ± 0.12^a^	ND
		Countertop (*n* = 3)	4.02 ± 0.50^a^	2.82 ± 0.30^a^	ND
		Package (*n* = 3)	ND	ND	ND
Raw materials and semifinished products in the production chain	Thawing	Meat (*n* = 3)	4.69 ± 1.43^a^	2.37 ± 0.04^ab^	ND
	Kneading	Meat (*n* = 4)	3.62 ± 0.20^ab^	1.09 ± 0.02^b^	ND
	Pickling	Meat (*n* = 16)	4.13 ± 0.84^a^	2.34 ± 0.98^ab^	1.44 ± 0.82
	Shaping	Meat (*n* = 3)	2.36 ± 0.62^b^	1.28 ± 0.51^b^	ND
	Pressing	Meat (*n* = 3)	4.06 ± 0.29^a^	3.50 ± 0.56^a^	ND
	Product	Meat (*n* = 6)	ND	ND	ND

n: total number of samples; ND means not detected in 10 g or per cm^2^. Different lowercase letters (a–c) within the same column signify significant differences among the samples (ANOVA, *P* < 0.05).

### 2.2 Cultivation of bacteria

For swab samples, the PBS was extracted from the cotton swabs into sterile Eppendorf tubes. A 1:10 dilution was prepared by transferring 100 μL of the eluate into 900 μL of PBS, followed by vortexing. Serial tenfold dilutions were subsequently prepared up to 10^–2^, 10^−3^, and 10^−4^. For meat and meatball samples, 10 g of each sample was aseptically weighed, mixed with 90 mL of buffered peptone water (BPW, Qingdao Hope Biol-Technology Co., Ltd., China), and homogenized in a stomacher at 25–30 °C for 2 min.

From appropriate dilutions, 100 μL aliquots were spread in duplicate onto Plate Count Agar (PCA, Qingdao Hope Biol-Technology Co., Ltd., China) and *Escherichia coli*/ coliform chromogenic agar plates (Qingdao Hope Biol-Technology Co., Ltd., China). Plates were incubated in an inverted position at 36 ± 2 °C for 24 h, with a minimum of three replicates per sample. After incubation, colonies on PCA were enumerated to determine total viable counts (TVCs), while coliform and *Escherichia coli* (*E. coli*) were identified based on colony color on the chromogenic plates.

### 2.3 Selection of sequencing samples

Based on the microbial contamination results summarized in Table 1, samples with the highest TVCs from both environmental and meat sources were selected for subsequent 16S rRNA gene sequencing. Elevated TVCs directly indicate compromised hygiene or active microbial proliferation, and sequencing these samples ensures capture of the microbial consortia most relevant to contamination risks. This selection prioritized samples with dense, diverse microbial communities ensuring sufficient genetic material for high resolution amplicon sequencing and those that best reflected contamination status at respective processing stages, as elevated TVCs typically indicate compromised hygiene or active microbial proliferation. Preliminary experiments confirmed these samples as optimal representatives of dominant microbial consortia. A total of 36 samples were analyzed, with detailed samples information provided in Supplementary Table S1.

### 2.4 DNA extraction and PCR amplification

For preprocessed samples, DNA were extracted using the OMEGA Soil DNA Kit (M5635-02) (Omega Bio-Tek, Norcross, GA, United States). The extracted DNA was subjected to 0.8% agarose gel electrophoresis to determine its molecular size, and Nanodrop was used for DNA quantification. For the bacterial project, the highly variable V3-V4 region of the bacterial 16S rRNA gene (approximately 480 bp in length) was selected for sequencing. PCR amplification was performed using specific primers targeting the V3-V4 region of the bacterial 16S rRNA gene: 341F (5′-CCTACGGGNGGCWGCAG-3′) and 806R (5′-GGACTACHVGGGTWTCTAAT-3′). The amplification products were analyzed by 2% agarose gel electrophoresis; the target fragment was excised and recovered using the magnetic bead sorting method. PCR products were quantified using the Quant-iT PicoGreen dsDNA Assay Kit on a Microplate Reader (BioTek, FLx800), followed by pooling of samples according to the required data volume for each sample.

### 2.5 Library preparation and sequencing

Library construction was performed using Illumina’s TruSeq Nano DNA LT Library Prep Kit. Libraries were quality-checked on an Agilent Bioanalyzer using the Agilent High Sensitivity DNA Kit (2100 assay). Libraries were quantified with the Quant-iT PicoGreen dsDNA Assay Kit on a Promega QuantiFluor; the calculated concentration of a qualified library should be above 2 nM. Qualified libraries were subjected to 2 × 250 bp paired-end sequencing on an Illumina NovaSeq platform using the NovaSeq 6000 SP Reagent Kit (500 cycles).

### 2.6 Statistical analysis

Bacterial counts were log_10_-transformed (CFU/g for meat, CFU/cm^2^ for environmental samples) prior to analysis. Means and standard deviations were calculated for each group. One-way ANOVA with post hoc tests (Tukey’s HSD) was used to evaluate differences among processing stages, and statistical assumptions (normality, homoscedasticity) were checked. Given the limited sample size (minimum n = 3), nonparametric and permutation-based methods were applied to verify robustness, yielding consistent results.

For six risk-associated bacterial taxa, relative abundance differences were evaluated using univariate tests on filtered, log-transformed data, with *p*-values adjusted for false discovery rate (FDR). Alpha diversity indices (Chao1 and Shannon) were computed using MicrobiomeAnalyst, and beta diversity was analyzed using Bray-Curtis distance matrices visualized via PCA and PCoA. Microbial source tracking was performed with SourceTracker, employing a modified Bayesian algorithm to estimate contributions from each processing stage and quantify known versus unassigned sources.

## 3 Results

### 3.1 Sanitary status of the processing environment and meat samples

**FIGURE 2:**
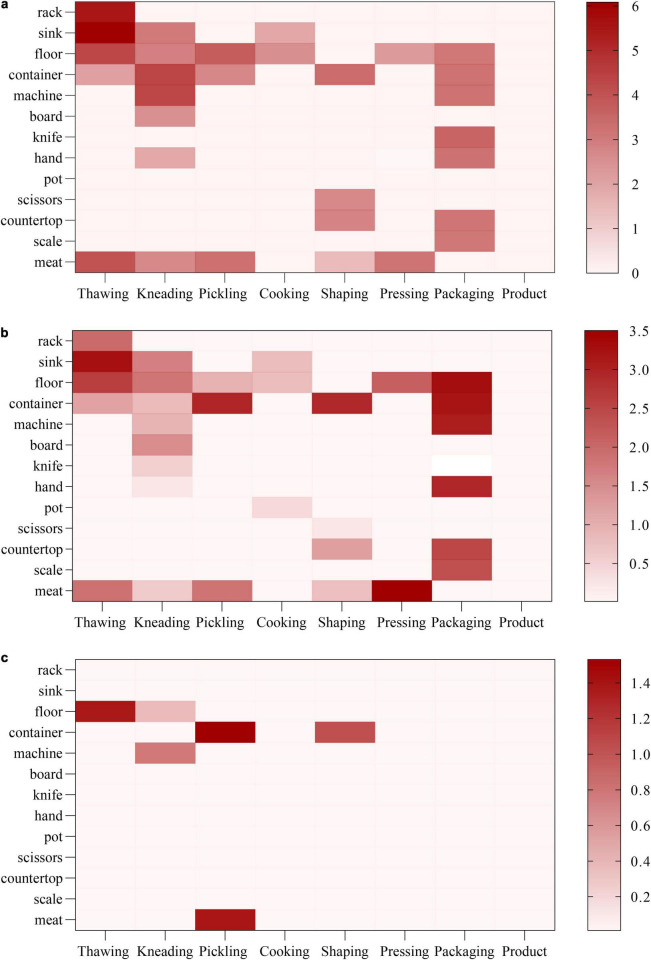
Sanitary conditions heatmap of samples in each technological process of Yao meat production, **(a)** Total viable counts; **(b)** Coliform; **(c)** *E. coli.*

From the perspective of environmental samples, the thawing stage posed the greatest sanitary risk. As shown in Table 1, the Total Viable Counts (TVCs) of sinks, racks, and floors at this stage reached 6.08 ± 0.30 log_10_ CFU/cm^2^, 5.71 ± 0.57 log_10_ CFU/cm^2^, and 4.90 ± 1.33 log_10_ CFU/cm^2^, respectively, with the coliform count in sinks as high as 3.35 ± 0.57 log_10_ CFU/cm^2^ all significantly higher than those of other environmental sites. The packaging stage also showed considerable contamination, excluding packaging bags, the TVCs of knives, machines, and countertops ranged from 3.97 ± 0.00 log_10_ CFU/cm^2^ to 4.38 ± 0.00 log_10_ CFU/cm^2^, while the average coliform count reached 3.51 ± 0.00 log_10_ CFU/cm^2^. Consistently, Figures 2a,b indicate that the thawing and packaging stages exhibited the highest levels of contamination, as reflected by the darker coloration compared with other production stages. As shown in Figure 2c, *E. coli* contamination was observed only sporadically, indicating its limited distribution and low abundance throughout the production chain. For meat samples, Figure 2a shows darker coloration for meat samples from the thawing, pickling, and pressing stages compared with shaping and final product stages, visually reflecting higher contamination levels.

### 3.2 Bacterial relative abundance based on taxonomic analysis

**FIGURE 3:**
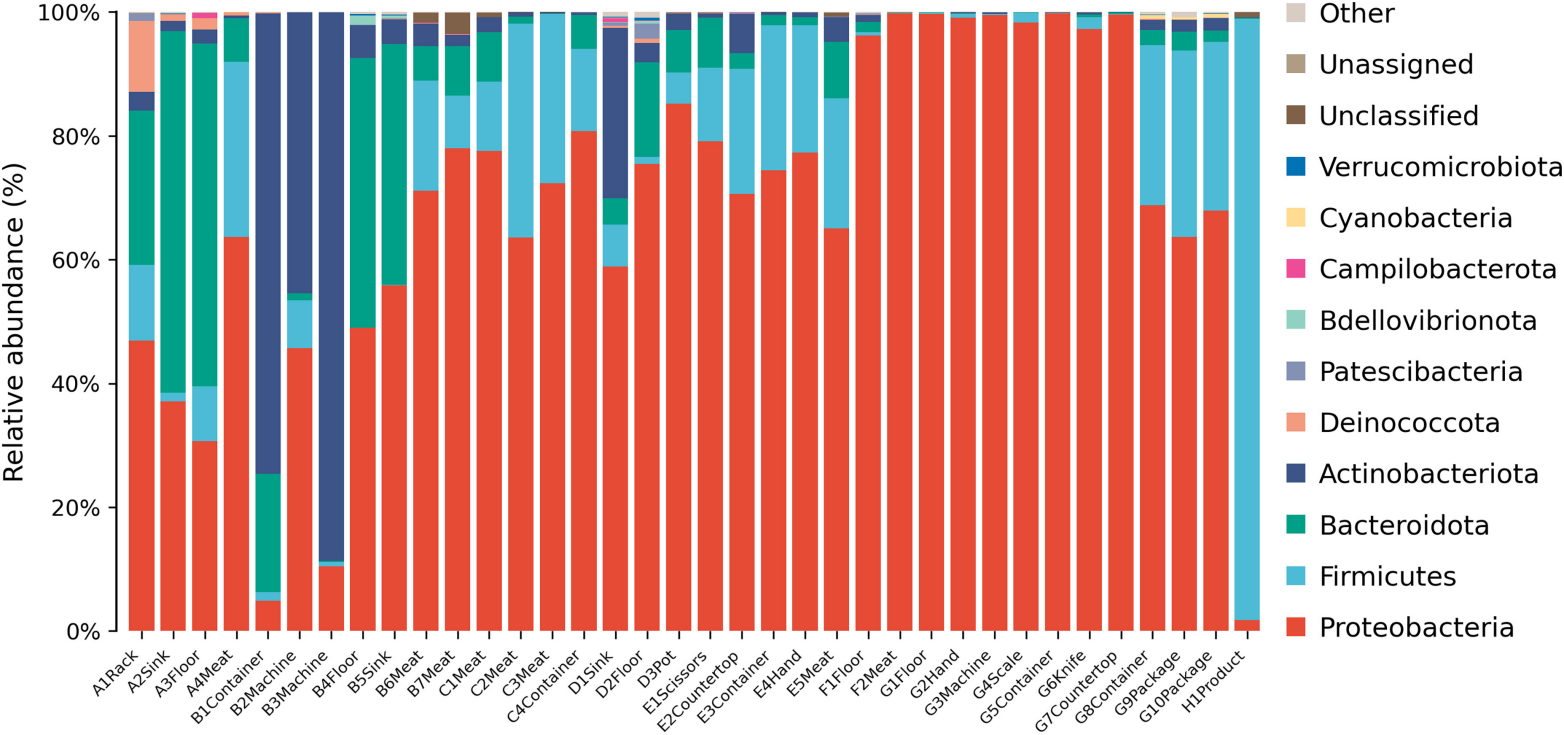
Relative abundance of microbes at the phylum level. A, Thawing; B, Kneading; C, Pickling; D, Cooking; E, Shaping; F, Pressing; G, Packaging; H, Product.

The microbial community at the phylum level varied significantly across different processing stages (Figure 3). Proteobacteria was the dominant phylum in most samples, and its abundance was particularly high during the packaging stage. Bacteroidota showed high abundances in the thawing and kneading stages but experienced a sharp decline after the cooking process. The pickling stage saw a significant increase in the abundance of Firmicutes, while Actinobacteriota had relatively high proportions during the kneading and shaping stages. Notably, Firmicutes hold an absolute dominant position in the microbial community in the final product.

**FIGURE 4:**
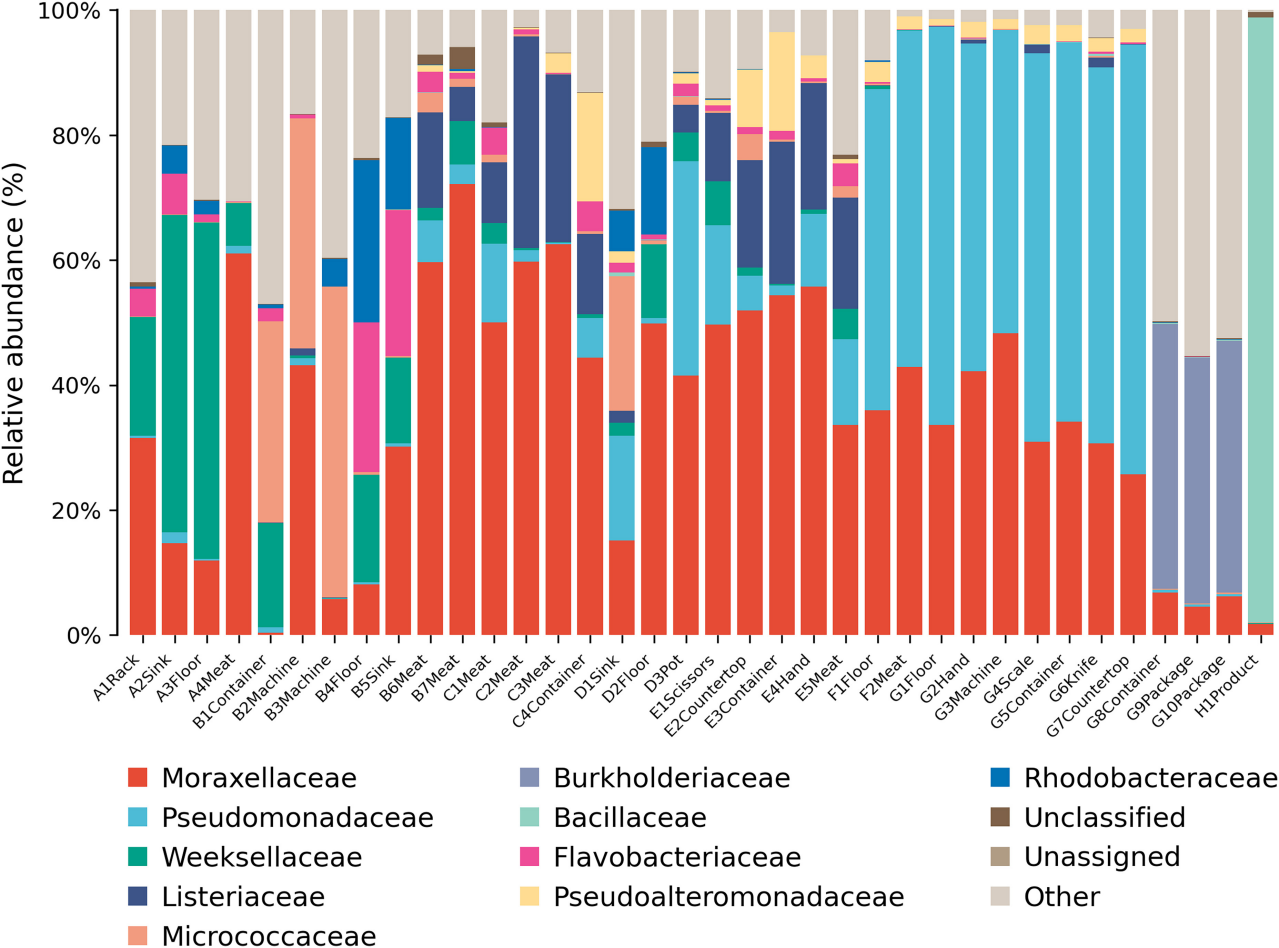
Relative abundance of microbes at the family level. A, Thawing; B, Kneading; C, Pickling; D, Cooking; E, Shaping; F, Pressing; G, Packaging; H, Product.

Moraxellaceae were widely distributed across various processing stages (Figure 4). Weeksellaceae dominated environmental samples in the thawing stage, and Micrococcaceae predominated in environmental samples from the kneading stage. Listeriaceae were detected in most samples from the pickling and shaping stages, especially meat samples. Pseudomonadaceae dominated during the pressing and packaging stages, and Bacillaceae dominated in the final product.

**FIGURE 5:**
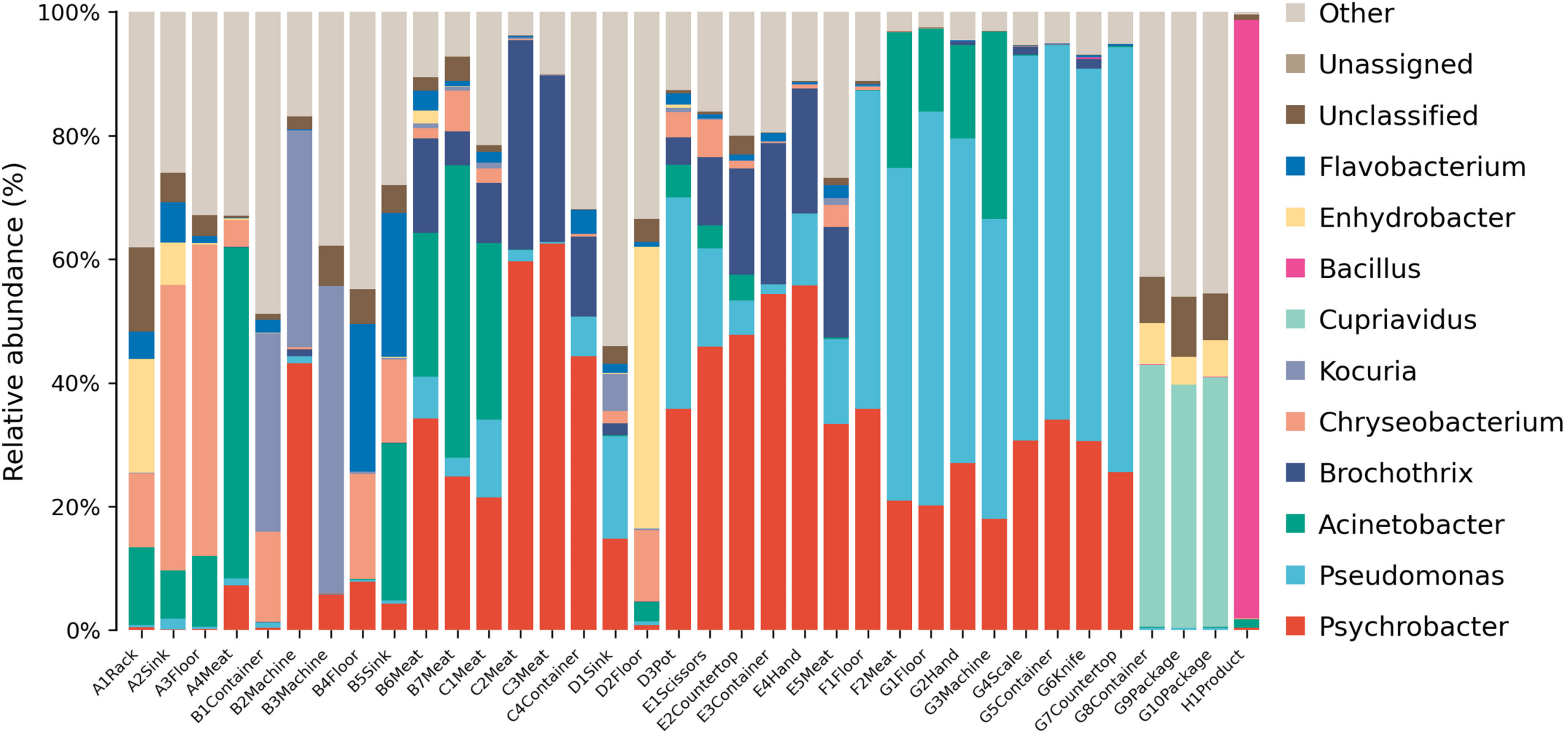
Relative abundance of microbes at the genus level present in the samples. A, Thawing; B, Kneading; C, Pickling; D, Cooking; E, Shaping; F, Pressing; G, Packaging; H, Product.

*Chryseobacterium* was detected in multiple samples, and its presence was particularly notable in the thawing stage (Figure 5). *Kocuria* accounted for a higher proportion of the microbial community in the meat contact sample during the kneading process. The pressing and packaging areas had similar microbial compositions, which were dominated by *Psychrobacter* and *Pseudomonas*. In the final product, the relative abundance of *Bacillus* was significantly higher, which was not detected in the previous stage.

#### 3.2.1 Bacterial composition and abundance

**FIGURE 6:**
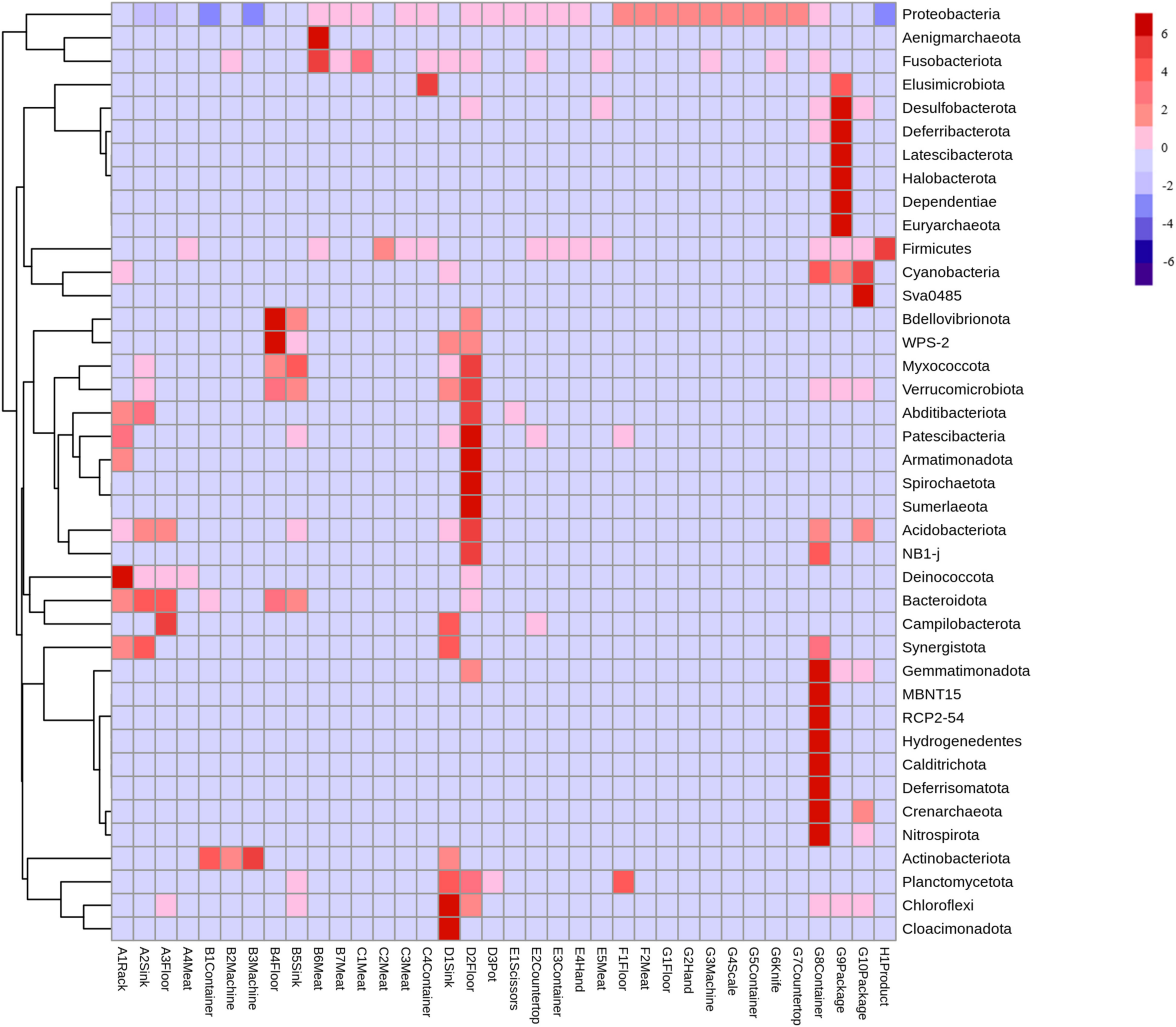
Heatmap plots of all samples at the phylum level. The color of each cell indicates the bacterial population: red signifies a high population, whereas blue denotes a low population. A, Thawing; B, Kneading; C, Pickling; D, Cooking; E, Shaping; F, Pressing; G, Packaging; H, Product.

At the phylum level, Deinococcota was detected during the thawing stage and dominated in the rack samples (Figure 6). The kneading and pickling stages had diverse bacterial communities, and Proteobacteria were more abundant in the meat samples. The sinks and floors during the cooking stage showed deep red in the heatmap, which indicates the proliferation of diverse bacteria. The pressing and packaging stages had concentrated bacterial communities, dominated by Proteobacteria, while only Firmicutes were prominent in the final product.

**FIGURE 7:**
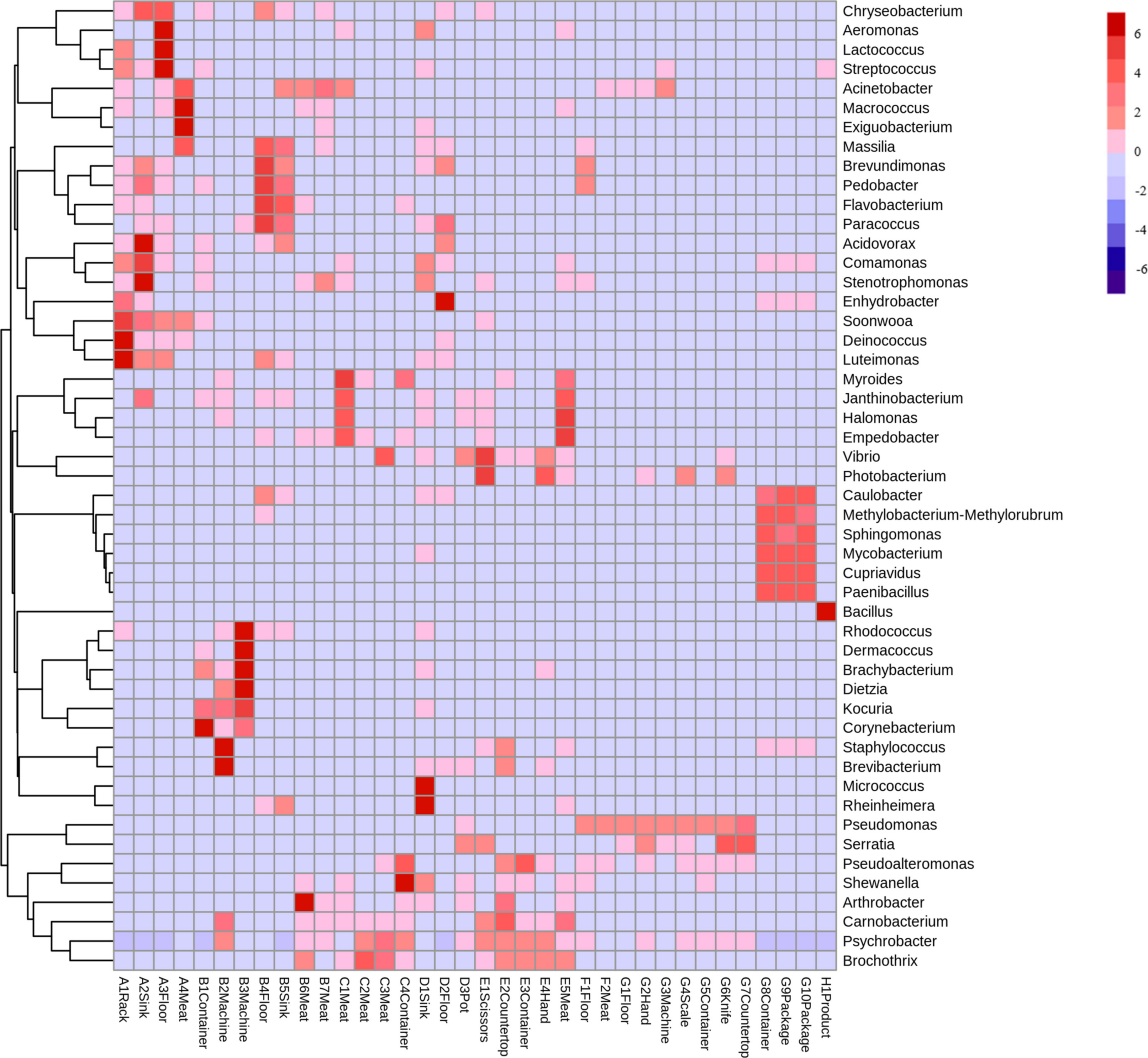
Heatmap plots of all samples at the genus level. The color of each cell indicates the bacterial population: red signifies a high population, whereas blue denotes a low population. A, Thawing; B, Kneading; C, Pickling; D, Cooking; E, Shaping; F, Pressing; G, Packaging; H, Product.

At the genus level, thawing stage samples had higher red block intensity, consistent with high total bacterial counts (Figure 7). *Soonwooa* was detected in all thawing samples, and *Psychrobacter* and *Brochothrix* were prevalent in most shaping stage samples. Bacterial abundance declined during pressing, with *Pseudomonas* becoming more dominant. In the final product, *Bacillus* showed the strongest red coloration, aligning with previous relative abundance data.

### 3.3 Bacterial correlation analysis

#### 3.3.1 Classical univariate test

**FIGURE 8:**
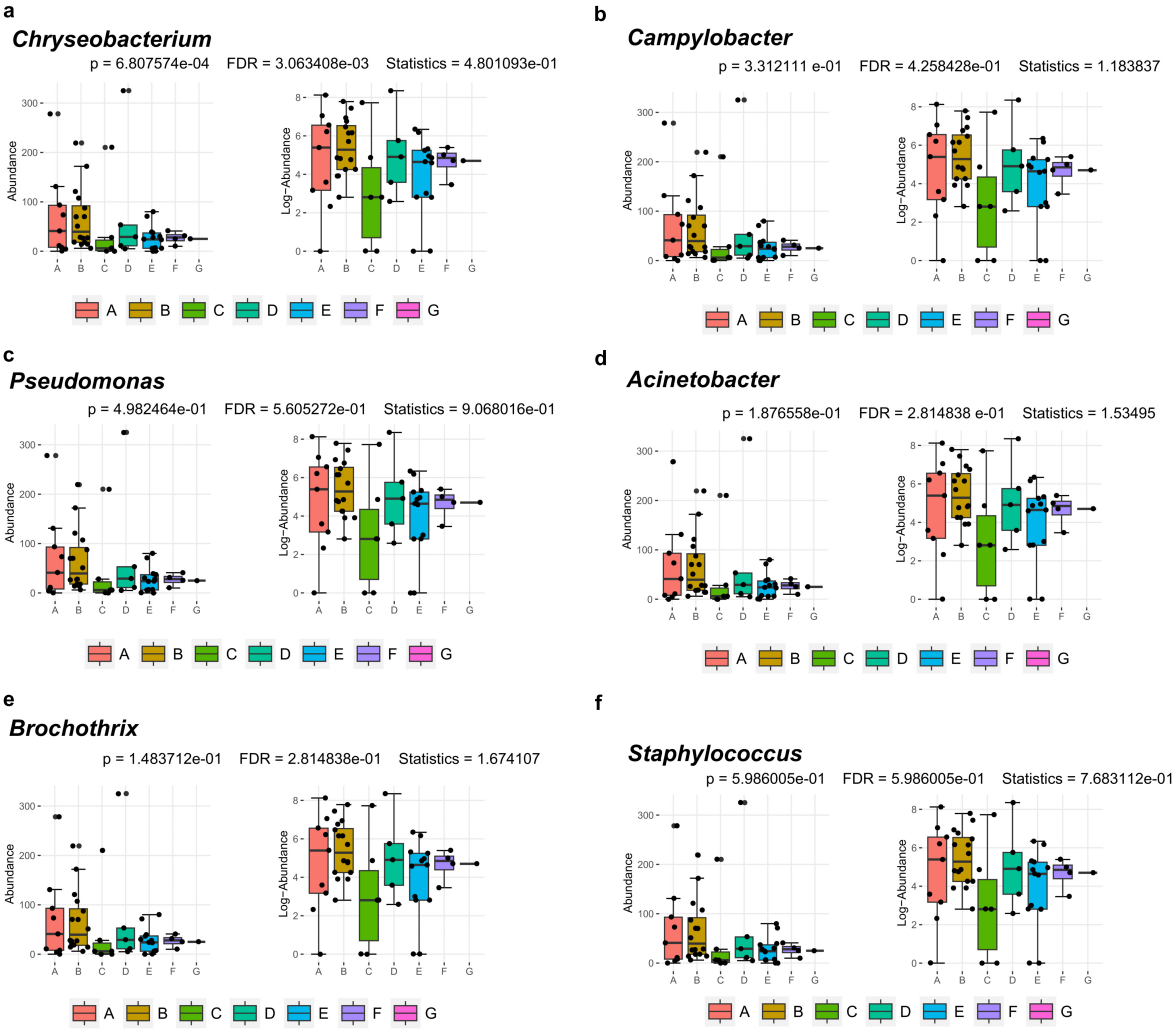
Filtered and log-transformed count of six pathogenic bacteria at seven stages in Yao Meat production line analyzed by univariate test. **(a)** *Chryseobacterium*. **(b)** *Campylobacter*. **(c)** *Pseudomonas*. **(d)** *Acinetobacter*. **(e)** *Brochothrix*. **(f)** *Staphylococcus*. A, Thawing; B, Kneading; C, Pickling; D, Cooking; E, Shaping; F, Pressing; G, Packaging.

16S rRNA sequencing has detected multiple potentially relevant spoilage indicator taxa linked to food spoilage and hygiene risks. Classical univariate statistical analysis was conducted to evaluate the relative abundance of six risk-associated bacterial taxa (Figure 8). As shown in the boxplot (Figure 8a), the relative abundance of *Chryseobacterium* varied significantly across production stages (*p = 6.81 × 10^−4^*). Its abundance was markedly higher during the thawing and kneading stages but declined sharply thereafter, suggesting that subsequent processing interventions effectively inhibited its growth. The other five pathogenic taxa like *Campylobacter* (Figure 8b), *Pseudomonas* (Figure 8c), *Acinetobacter* (Figure 8d), *Brochothrix* (Figure 8e), and *Staphylococcus* (Figure 8f) exhibited distinct fluctuations in relative abundance across processing stages but these variations did not reach statistical significance (*p > 0.05*).

#### 3.3.2 Diversity analysis

**FIGURE 9:**
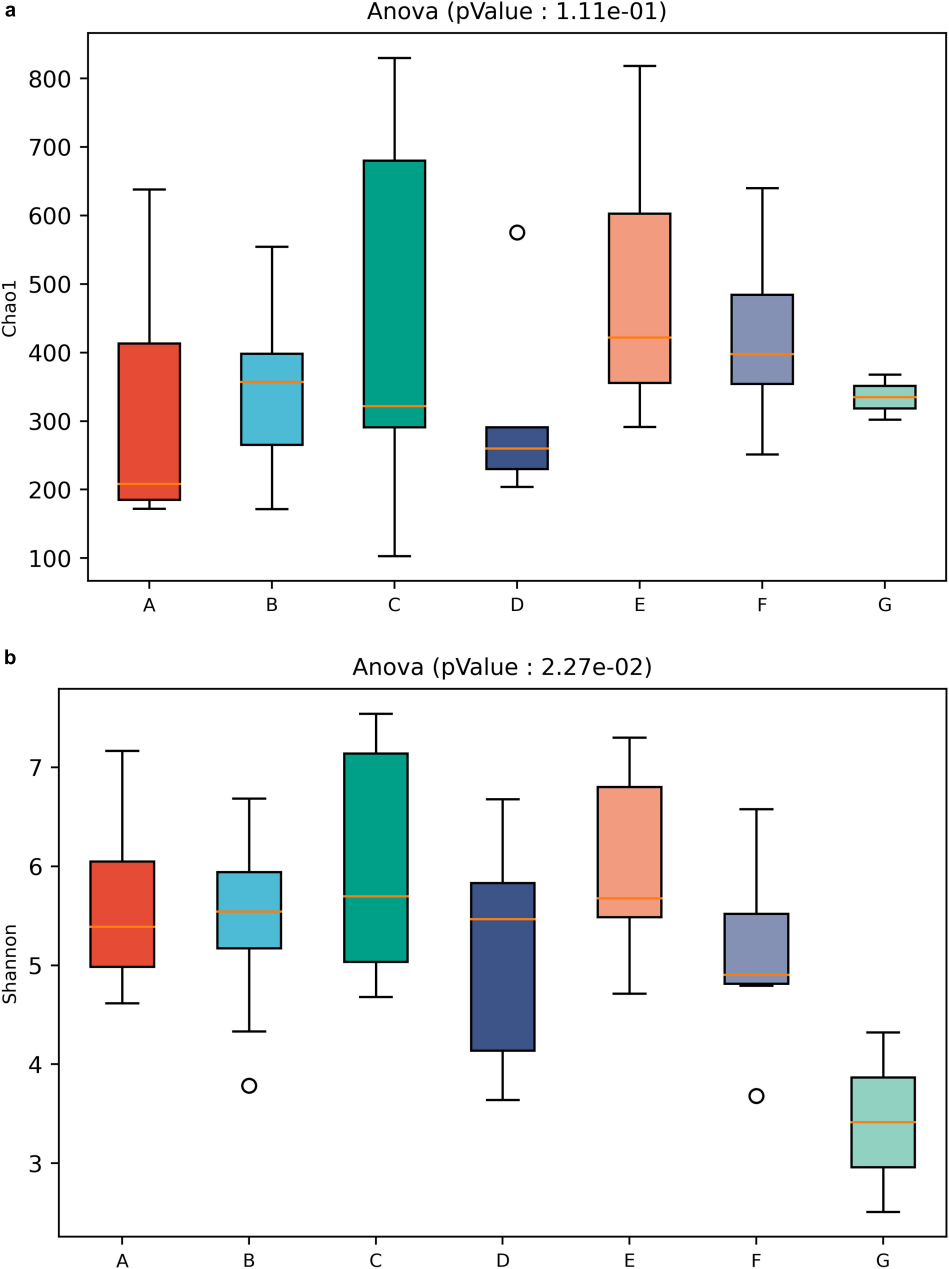
Alpha diversity at genus level for all samples from each processing, using **(a)** Chao 1 index and **(b)** Shannon index. A, Thawing; B, Kneading; C, Pickling; D, Cooking; E, Shaping; F, Pressing; G, Packaging.

**FIGURE 10:**
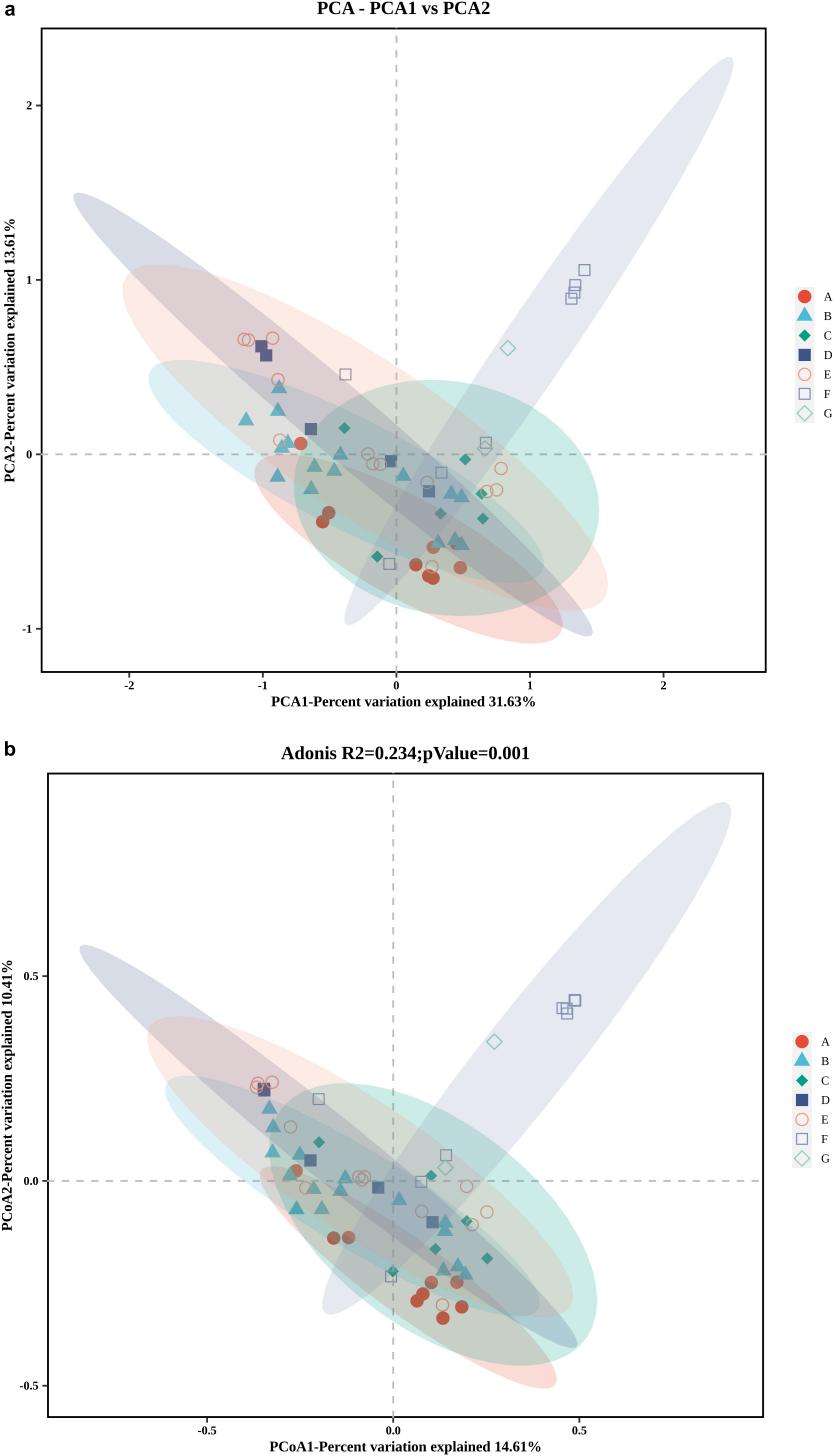
Beta diversity analysis of microbial communities during the production of Yao meat. **(a)** Beta diversity at PCA based on Bray-Curtis algorithm; **(b)** Beta diversity at PCoA based on Bray-Curtis Algorithm. A, Thawing; B, Kneading; C, Pickling; D, Cooking; E, Shaping; F, Pressing; G, Packaging.

At the genus level, samples from the kneading, pickling, and cooking stages had high alpha diversity (Figure 9). Both Chao1 and Shannon indices were elevated during pickling. Principal Component Analysis (PCA) and Principal Coordinate Analysis (PCoA) assessed bacterial community beta diversity (Figure 10). With cooking as a dividing point, the first three and latter three processing stages were clearly separated. There are differences in beta diversity among the three stages of shaping, pressing, and packaging.

### 3.4 Determining the bacterial contribution of sources

**FIGURE 11:**
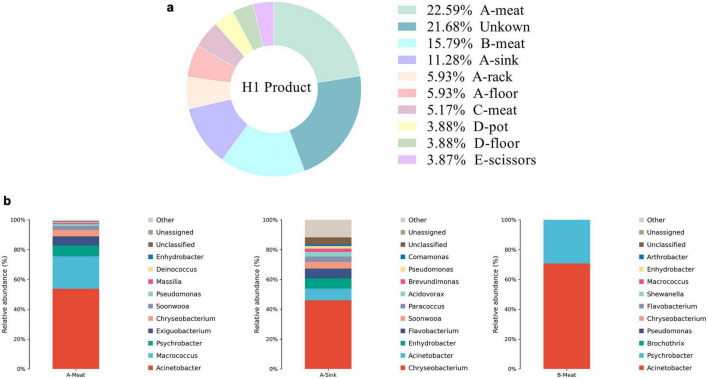
Contributions of microbial propagation sources at the genus level. A, Thawing; B, Kneading; C, Pickling; D, Cooking; E, Shaping. **(a)** Source contribution proportion of the microbial community in the final product of Yao Meat. **(b)** Microbial community composition of key contamination stages of Yao meat.

Genus level source tracking identified the origins of the final product’s microbial community (Figure 11). Known contamination sources accounted for 78.32% of the microbiota, while 21.68% remained unassigned (Figure 11a). The thawing stage was the largest contributor: thawing sinks (11.28%, dominated by *Chryseobacterium*) and thawed meat (22.59%, high in *Acinetobacter*, *Macrococcus*, and *Psychrobacter*) (Figures 11a,b). The kneading stage also contributed substantially, with kneaded meat accounting for 15.79% (primarily *Acinetobacter* and *Psychrobacter*). Additional contributions came from pickled meat (5.17%), cooking pots and cooking area floors (3.88%), and shaping scissors (3.88%).

## 4 Discussion

Our findings that RTE meat processing environments harbor persistent pathogens and are susceptible to post-cooking contamination are consistent with previous studies on RTE meat products (Beccalli et al., 2019). Notably, our culture-based analyses only quantified common hygiene indicators and did not include targeted detection of major RTE meat pathogens. By employing 16S rRNA gene sequencing, this study overcame the limitations of traditional methods, systematically characterized the microbial dynamics throughout the entire production process of RTE products for the first time, and identified critical points for contamination control. Microbial source tracking further elucidated the origins of contamination in the final product. These results can inform the development of targeted strategies to mitigate contamination in traditional RTE meat production. The research results, together with the characteristics of microbial community changes during the refrigeration process of Yao meat (Zhang et al., 2018), will provide data support for the reduction of pollution for RTE meat.

### 4.1 Microbial contamination status in key processing stages

The thawing stage was identified as a critical control point, with high bacterial loads detected (Table 1) and highly potentially relevant spoilage indicator taxa (Figure 8). This is consistent with the biological properties of frozen meat: dormant microorganisms recover metabolic activity during thawing, while exudates promote microbial proliferation (Leygonie et al., 2012). Based on this understanding, we propose that the observed contamination could be associated with factors such as (i) thawing at room temperature, which exceeds the recommended 0–4 °C and may facilitate microbial growth, and (ii) prolonged thawing time (8–10 h), which may further increase microbial proliferation. However, these explanations remain hypothetical and require further experimental validation. This contamination situation is consistent with observations in processed meat production, where pre-heating stages of mixed pressed ham and cooked sausage exhibited significant microbial enrichment resulting from inadequate temperature control (Park et al., 2014). According to the Chinese National Food Safety Standard for Fresh (Frozen) Livestock and Poultry Products (GB 2707–2016) China, 2016, the permissible limits for frozen pork are 6.00 log_10_ CFU/g for TVCs and 4.00 log_10_ CFU/g for coliforms. Although the TVC of thawed meat (4.69 ± 1.43 log_10_ CFU/g) did not exceed this limit, its coliform count approached the threshold, indicating an initial contamination risk. Optimization of thawing practices is therefore essential, with alternatives such as low-temperature or plasma activated water thawing recommended to mitigate microbial risks (Liao et al., 2020; Qian et al., 2022; Wang et al., 2012). Meanwhile, emerging thawing technologies can be introduced into the factory. Relative to conventional thawing approaches, both PAW thawing and ultrasound PAW combined thawing brought about a 0.62–1.17 log CFU/g reduction in bacterial counts (Qian et al., 2022). Ultrasonic thawing can inhibit microbial growth to extend the shelf life and quality of food, and this emerging technology has been used as a thawing solution for traditional food processing (Liao, 2022). Ohmic thawing can shorten the thawing time and reduce microbial growth (Döner et al., 2024). By optimizing thawing technology, the microorganisms in the thawing process can be controlled.

The resurgence of microbial loads during the packaging stage (Table 1) highlights persistent sanitation gaps in this final processing step. Similar findings have been reported in previous studies, where insufficient sterilization-due to equipment malfunction, inadequate disinfection protocols, or poor implementation of hygiene practice-led to post-cooking contamination (Seavey, 2016). Although cooking reduced microbial counts, the absence of a post-pressing heating step meant that contamination during pressing posed a direct safety risk. Stricter control during pressing is necessary, including enhanced disinfection of pressing and packaging equipment. In addition, the production of Yao meat requires the addition of spices. There may be unknown microbial contamination in spices, therefore, testing and pasteurization of spices should be incorporated at the kneading stage to reduce the introduction of pathogenic taxa (Nei et al., 2015). As spices were not included in the sampling scheme, their contribution to contamination cannot be determined and any inference remains speculative. Further studies are needed to evaluate whether spices act as a source of microbial contamination during processing. For the spices used in the kneading stage, propylene oxide, controlled condensation pasteurization technology, radio frequency, and infrared treatments can be used to maintain the original flavor of the spices while meeting the sterilization effect (Pan et al., 2012).

### 4.2 Taxonomic characteristics and potentially relevant spoilage indicator taxa

Lack of cleanliness in the kitchen can lead to diverse microbial populations (Flores et al., 2013). Proteobacteria dominated most processing stages, particularly packaging (Figures 3, 6), consistent with their prevalence in meat and vegetable processing environments (Jiang et al., 2023; Yang et al., 2018). This phylum includes many spoilage and pathogenic taxa (e.g., *Pseudomonas*, *Acinetobacter*) that threaten product safety. The sharp decline in Bacteroidota after cooking reflects their heat sensitivity (Yin et al., 2022), validating the effectiveness of cooking. In contrast, Firmicutes predominated in the final product (97.2%), in line with reports of its dominance in fresh and cured meats (Hwang et al., 2020; Wang et al., 2021). Within Firmicutes, Bacillaceae emerged as key spoilage organisms (Zhang et al., 2018). Some *Bacillus* species are known to cause food spoilage and, in some cases, foodborne illness (Fernández-No et al., 2011). Although genus-level 16S rRNA sequencing cannot confirm species-level pathogenicity, the high relative abundance of this genus suggests a potential food hygiene risk.

At finer taxonomic levels, several groups presented specific risks. Listeriaceae, detected in the pickling and shaping stages (Figure 4), persist in meat processing environments due to their cold tolerance, disinfectant resistance, and strong surface adhesion (Salvat et al., 1995). Although *Listeria monocytogenes* was not specifically investigated in this study, its potential presence cannot be excluded, highlighting a possible hygiene concern and underscoring the importance of strict raw material control and thorough sanitation of brining equipment (Bērzi ,nš et al., 2010; Thévenot et al., 2006). *Pseudomonadaceae*, prevalent in pressing and packaging (Figure 4), are widely recognized spoilage bacteria (Chen et al., 2024), capable of forming resilient biofilms on stainless steel (Tirpanci Sivri et al., 2023). Their psychrotrophic nature makes them especially problematic in cold stages, emphasizing the need for biofilm-targeted disinfection (Nikolaev et al., 2022). *Chryseobacterium* showed high relative abundance (Figures 5, 7) and was significantly enriched during the thawing and kneading stages (Figure 8a). This genus, associated with frozen meat spoilage (Olofsson et al., 2007) and opportunistic infections (Mwanza et al., 2022), underscores the need for improved raw material screening and environmental sanitation. As only the highest-TVC samples were sequenced, the relative abundance of these taxa may be slightly overestimated, reflecting the most contaminated microenvironments rather than the entire production environment.

### 4.3 Microbial diversity dynamics and contamination source attribution

High alpha diversity in kneading, pickling, and cooking stages (Figures 9a,b) indicates complex microbial communities introduced by spices or environmental exposure. Elevated Chao1 and Shannon indices during pickling suggest both flavor-related microbial activity (Byrne et al., 2002), and contamination risks from spices, underscoring the need for stringent quality control of seasoning ingredients. The diversity decline after cooking highlights the effectiveness of thermal inactivation. Beta diversity analysis further demonstrated that cooking is the link that causes changes in microbial communities (Figures 10a,b).

The findings of Source tracking emphasize the need to optimize thawing conditions and improve sanitation of kneading equipment. *Chryseobacterium* which is a psychrotrophic bacterium linked to frozen meat spoilage flourishes in the thawing stage due to room-temperature thawing and accumulated meat exudates, which provide rich nutrients for its growth (Olofsson et al., 2007). *Acinetobacter* and *Psychrobacter* persist in thawed meat because raw frozen pork lacks sufficient pre-thaw microbial control, allowing these adhesion-prone genera to carry over to subsequent steps. In kneading, Acinetobacter forms biofilms in equipment crevices (Nikolaev et al., 2022) that evade routine cleaning, while *Psychrobacter* transfers from cold processing environments to meat via contaminated surfaces both driving cross contamination. Even small sources can pose residual risks. Notably, Bacillus, dominant in the final product, was not detected in any tracked sources, suggesting it may originate from unmeasured sources or reflect source-tracking limitations. The detection of *Bacillus* poses a threat to subsequent preservation, as this genus can grow slowly at refrigerated temperatures (Soni et al., 2018). This finding also provides a key basis for improving postpartum storage plans.

A proportion of the microbiota (21.68%) remained unassigned, suggesting contributions from unmeasured sources or methodological limitations. Notably, Bacillus, which dominated the final product, was not detected in any tracked sources, indicating it may originate from unsampled reservoirs, spore persistence, or low-abundance taxa below detection limits. In addition, the limited sample size may have constrained source identification. Future studies should expand sampling coverage and apply higher-resolution approaches to improve traceability.

## 5 Conclusion

In this study, the microbial communities across the entire processing chain of Yao meat were characterized using traditional microbial culture techniques and 16S rRNA gene sequencing technology. The results revealed that the thawing stage was a potential contamination point. Proteobacteria predominated during processing stages, while Firmicutes became the dominant phylum in the microbial community of the final product. Additionally, this study detected taxa closely associated with product spoilage at various processing stages; among these, the genus *Chryseobacterium* exhibited a notably high abundance during the thawing stage. Source tracking of the final product showed that 78.32% of its microbiota originated from known contamination points, while the source of the remaining 21.68% remained unassigned. The genus *Bacillus* was exclusively detected in the final product, but absent from the tracked sources, suggesting that the traceability scope needs to be expanded to effectively mitigate contamination risks. Our research findings fill the gap in microbial control throughout the entire production chain of Vacuum Packed Yao Meat. We put forward valuable recommendations for controlling critical contamination points during production, which not only help improve the safety and quality of Vacuum Packed Yao Meat but also provide a basis for optimizing microbial control in the entire RTE meat processing system. In future studies, targeted pathogen detection will be incorporated to better evaluate the food safety relevance of the identified contamination pathways and to strengthen risk characterization.

## Data Availability

The datasets presented in this study can be found in online repositories. The names of the repository/repositories and accession number(s) can be found in the article/Supplementary material.
