# Supplementary material for: Dynamics of microbial contamination and hygiene risk points in the production of ready-to-eat Yao meat
Source: Front Microbiol. 2026 Apr 22;17:1829503. doi: 10.3389/fmicb.2026.1829503 (PMC13144047; doi:10.3389/fmicb.2026.1829503)
Supplement: Supplementary file 1 [file Supplementary_file_1.docx]

Table S1. Selected samples for further high-throughput 16S rRNA analysis.

| **Sample name** | **Processing** | **Accessed number** |
| --- | --- | --- |
| A1Rack | Thawing | [SRR35738339](https://dataview.ncbi.nlm.nih.gov/object/SRR35738339) |
| A2Sink | Thawing | [SRR35738338](https://dataview.ncbi.nlm.nih.gov/object/SRR35738338) |
| A3Floor | Thawing | [SRR35738327](https://dataview.ncbi.nlm.nih.gov/object/SRR35738327) |
| A4Meat | Thawing | [SRR35738316](https://dataview.ncbi.nlm.nih.gov/object/SRR35738316) |
| B1Container | Kneading | [SRR35738309](https://dataview.ncbi.nlm.nih.gov/object/SRR35738309) |
| B2Machine | Kneading | [SRR35738308](https://dataview.ncbi.nlm.nih.gov/object/SRR35738308) |
| B3Machine | Kneading | [SRR35738307](https://dataview.ncbi.nlm.nih.gov/object/SRR35738307) |
| B4Floor | Kneading | [SRR35738306](https://dataview.ncbi.nlm.nih.gov/object/SRR35738306) |
| B5Sink | Kneading | [SRR35738305](https://dataview.ncbi.nlm.nih.gov/object/SRR35738305) |
| B6Meat | Kneading | [SRR35738304](https://dataview.ncbi.nlm.nih.gov/object/SRR35738304) |
| B7Meat | Kneading | [SRR35738337](https://dataview.ncbi.nlm.nih.gov/object/SRR35738337) |
| C1Meat | Pickling | [SRR35738336](https://dataview.ncbi.nlm.nih.gov/object/SRR35738336) |
| C2Meat | Pickling | [SRR35738335](https://dataview.ncbi.nlm.nih.gov/object/SRR35738335) |
| C3Meat | Pickling | [SRR35738334](https://dataview.ncbi.nlm.nih.gov/object/SRR35738334) |
| C4Container | Pickling | [SRR35738333](https://dataview.ncbi.nlm.nih.gov/object/SRR35738333) |
| D1Sink | Cooking | [SRR35738332](https://dataview.ncbi.nlm.nih.gov/object/SRR35738332) |
| D2Floor | Cooking | [SRR35738331](https://dataview.ncbi.nlm.nih.gov/object/SRR35738331) |
| D3Pot | Cooking | [SRR35738330](https://dataview.ncbi.nlm.nih.gov/object/SRR35738330) |
| E1Scissors | Shaping | [SRR35738329](https://dataview.ncbi.nlm.nih.gov/object/SRR35738329) |
| E2Countertop | Shaping | [SRR35738328](https://dataview.ncbi.nlm.nih.gov/object/SRR35738328) |
| E3Container | Shaping | [SRR35738326](https://dataview.ncbi.nlm.nih.gov/object/SRR35738326) |
| E4Hand | Shaping | [SRR35738325](https://dataview.ncbi.nlm.nih.gov/object/SRR35738325) |
| E5Meat | Shaping | [SRR35738324](https://dataview.ncbi.nlm.nih.gov/object/SRR35738324) |
| F1Floor | Pressing | [SRR35738323](https://dataview.ncbi.nlm.nih.gov/object/SRR35738323) |
| F2Meat | Pressing | [SRR35738322](https://dataview.ncbi.nlm.nih.gov/object/SRR35738322) |
| G1Floor | Packaging | [SRR35738321](https://dataview.ncbi.nlm.nih.gov/object/SRR35738321) |
| G2Hand | Packaging | [SRR35738320](https://dataview.ncbi.nlm.nih.gov/object/SRR35738320) |
| G3Machine | Packaging | [SRR35738319](https://dataview.ncbi.nlm.nih.gov/object/SRR35738319) |
| G4Scale | Packaging | [SRR35738318](https://dataview.ncbi.nlm.nih.gov/object/SRR35738318) |
| G5Container | Packaging | [SRR35738317](https://dataview.ncbi.nlm.nih.gov/object/SRR35738317) |
| G6Knife | Packaging | [SRR35738315](https://dataview.ncbi.nlm.nih.gov/object/SRR35738315) |
| G7Countertop | Packaging | [SRR35738314](https://dataview.ncbi.nlm.nih.gov/object/SRR35738314) |
| G8Container | Packaging | [SRR35738313](https://dataview.ncbi.nlm.nih.gov/object/SRR35738313) |
| G9Package | Packaging | [SRR35738312](https://dataview.ncbi.nlm.nih.gov/object/SRR35738312) |
| G10Package | Packaging | [SRR35738311](https://dataview.ncbi.nlm.nih.gov/object/SRR35738311) |
| H1Product | Product | [SRR35738310](https://dataview.ncbi.nlm.nih.gov/object/SRR35738310) |

https://www.ncbi.nlm.nih.gov/bioproject/PRJNA1337805/
